# Case presentation: T9-T11 transverse myelitis with double viral etiology in an immunocompetent host

**DOI:** 10.1186/1471-2334-14-S7-P12

**Published:** 2014-10-15

**Authors:** Ramona Ștefania Popescu, Adina Ilie, Adrian Streinu-Cercel

**Affiliations:** 1 Carol Davila University of Medicine and Pharmacy, Bucharest, Romania; 2 National Institute for Infectious Diseases "Prof. Dr. Matei Balş", Bucharest, Romania

## Background

Transverse myelitis is a neurological disorder characterized by spinal cord inflammation, which can associate myelin damage, resulting in varying degrees of weakness, sensory alterations, and autonomic dysfunction. Some of the infectious etiologies include herpes viruses, influenza virus, echovirus, HIV, hepatitis A and rubella viruses.

## Case report

We report the case of a 33-year-old woman who developed acute transverse myelitis with cerebrospinal fluid (CSF) findings suggestive for a central nervous system (CNS) infection with positive serology for acute herpes simplex virus-2 (HSV-2) infection and cytomegalovirus (CMV) DNA detection in CSF.

This immunocompetent female patient presented with acute urine retention, complete absence of stool or gas and saddle paresthesia irradiated on the front of her lower limbs. Her spinal magnetic resonance imaging findings, CSF analysis and clinical picture were compatible with transverse myelitis. She also had skin rash resembling herpetic infection and her blood serology was positive for acute HSV-2 infection. She received treatment with IV acyclovir with partial resolution of the symptoms. Because of the persistence of symptoms for over 1 year and the presence of CMV IgG (previously negative) we performed further investigation that revealed positive PCR for CMV from the CSF. We initiated treatment with ganciclovir IV with good clinical response.

After more than 1 year from the first symptoms, the patient is still under close supervision of the neurologist and the infectious diseases doctor, with almost total recovery, accusing only mild paresthesia and weakness in the lower limbs during effort.

## Conclusion

The isolation of CMV in the CSF suggests a direct viral toxicity implicated in the pathogenesis of transverse myelitis but we still have to take into consideration an immunological mechanism due to HSV-2 infection. This case highlights the fact that double viral etiology is very rare, but possible and that these viruses may cause serious infections even in an immunocompetent host.

